# Internalization of isolated functional mitochondria: involvement of macropinocytosis

**DOI:** 10.1111/jcmm.12316

**Published:** 2014-06-09

**Authors:** Tomoya Kitani, Daisuke Kami, Satoaki Matoba, Satoshi Gojo

**Affiliations:** a Department of Cardiovascular Medicine, Graduate School of Medical Science, Kyoto Prefectural University School of MedicineKyoto, Japan; b Department of Regenerative Medicine, Graduate School of Medical Science, Kyoto Prefectural University of MedicineKyoto, Japan

**Keywords:** mitochondrial transfer, rho0 cells, macropinocytosis

## Abstract

In eukaryotic cells, mitochondrial dysfunction is associated with a variety of human diseases. Delivery of exogenous functional mitochondria into damaged cells has been proposed as a mechanism of cell transplant and physiological repair for damaged tissue. We here demonstrated that isolated mitochondria can be transferred into homogeneic and xenogeneic cells by simple co-incubation using genetically labelled mitochondria, and elucidated the mechanism and the effect of direct mitochondrial transfer. Intracellular localization of exogenous mitochondria was confirmed by PCR, real-time PCR, live fluorescence imaging, three-dimensional reconstruction imaging, continuous time-lapse microscopic observation, flow cytometric analysis and immunoelectron microscopy. Isolated homogeneic mitochondria were transferred into human uterine endometrial gland-derived mesenchymal cells in a dose-dependent manner. Moreover, mitochondrial transfer rescued the mitochondrial respiratory function and improved the cellular viability in mitochondrial DNA-depleted cells and these effects lasted several days. Finally, we discovered that mitochondrial internalization involves macropinocytosis. In conclusion, these data support direct transfer of exogenous mitochondria as a promising approach for the treatment of various diseases.

## Introduction

Cell-based therapies for organ regeneration are currently emerging as a new promising approach to treat various diseases, including cardiovascular and neurodegenerative diseases [1–3]. However, the mechanism supporting these therapeutic effects remains poorly understood. Several studies indicated that the intercellular transfer of organelles, including mitochondria, might contribute to these effects [4–6].

Mitochondria are considered cellular ‘power plants’ because they largely synthesize the universal energy ‘currency’ of the cells, *i.e*. adenosine triphosphate (ATP). In addition, mitochondrial dysfunction is associated with many diseases, including metabolic and neurodegenerative disorders [7,8]. *In vitro* experiments revealed that mitochondrial DNA (mtDNA)-depleted mammalian cells (ρ0 cells) recovered aerobic respiration after intercellular mitochondrial transfer from intact cells [6]. Furthermore, an *in vitro* model of ischaemia was successfully used to rescue injured cardiomyoblasts from cell death through direct cell-to-cell interaction involving mitochondrial transfer [5]. Few studies reported that the culture of mammalian cells with isolated mitochondria resulted in mitochondrial internalization [9,10]. However, other reports were unable to detect the cellular internalization of isolated mitochondria during simple co-incubation [6,11]. Nonetheless, the therapeutic potential of this approach was supported by an *in vivo* study conducted on rabbit model of myocardial infarction [12,13]. Direct injection of autologous mitochondria into the ischaemic heart considerably increased the tissue ATP content and improved post-infarct cardiac functions. It has also been shown in *in vitro* studies that a large number of isolated mitochondria were taken up by cardiomyocytes after a 24-hour co-incubation. In addition, xenogeneic mitochondria were also used to discriminate between native and transplanted mitochondria. However, *in vivo*, the majority of the transplanted mitochondria remained in the interstitial space and only a limited number were internalized into the cells. Taken altogether, these studies emphasize that the current internalization protocols for isolated mitochondria remain inefficient, most likely because of our poor understanding of the endogenous mechanism.

We here demonstrated and monitored the mitochondrial transfer into mammalian cells by using genetically labelled mitochondria. We also assessed the impact of the transfer on the mitochondrial function and viability of the cells. Finally, the mechanism of mitochondrial internalization was investigated by using endocytosis inhibitors.

## Materials and methods

### Cell culture

The H9c2 cardiomyoblasts were obtained from American Type Culture Collection (Rockville, MD, USA). Human uterine EMCs were kindly provided by Dr. Umezawa [14]. The H9c2 cells stably expressing green fluorescent protein (GFP) were generated with a recombinant retrovirus carrying GFP driven by the pMSCV-puro retroviral vector. Mito-DsRed2 vectors (Clontech, Palo Alto, CA, USA) were digested with restriction enzymes and inserted into the pMX retroviral vector. EMCs-DsRed2 mito were prepared as described previously [15] and purified by fluorescence-activated cell sorting (FACS) 1 week after retrovirus transfection. The H9c2 cells, EMCs and EMCs-DsRed2 mito were maintained in DMEM (Life Technologies, Tokyo, Japan) supplemented with 10% foetal bovine serum (Life Technologies) and 1% penicillin/streptomycin (Life Technologies; standard medium). The H9c2 cells stably expressing GFP were maintained in standard medium containing 1 μg/ml puromycin. The ρ0 cells were generated as described previously and cultured in ρ0 medium (standard medium with 110 μg/ml pyruvate (Life Technologies) and 50 μg/ml uridine (Sigma-Aldrich, Tokyo, Japan) [16]. All cell lines were incubated at 37°C under 5% CO_2_. All experiments were conducted with cultures at ∼80% confluence.

### Mitochondrial isolation and transfer

Mitochondria were isolated from the EMCs-DsRed2 mito by differential centrifugation. The cells were harvested from culture dishes with homogenization buffer [HB; 20 mM HEPES-KOH (pH 7.4), 220 mM mannitol and 70 mM sucrose] containing a protease inhibitor mixture (Sigma-Aldrich) and pelleted by centrifugation (2300 × g; 5 min.). The cell pellet was resuspended in HB and incubated on ice for 5 min. All of the following steps were conducted at 4°C. The cells were ruptured by 10 strokes of a 27-gauge needle. The homogenate was centrifuged (400 × g; 5 min.) two times to remove unbroken cells. The mitochondria were harvested by centrifugation (5800 × g; 5 min.) and resuspended in HB. The amount of isolated mitochondria was expressed as protein concentration by using a Bio-Rad protein assay kit (Bio-Rad, Richmond, CA, USA). The isolated mitochondria were resuspended in 1 ml of HB for their characterization. First, the hydrodynamic size and surface charge (zeta potential: electrostatic potential generated by the accumulation of ions at the surface of colloidal particles) of isolated mitochondria were determined by dynamic light scattering and electrophoretic light scattering measured by using a Zetasizer Nano ZS (Malvern Instruments, Malvern, UK; triplicate) [17].

Mitochondrial transfer was conducted by co-incubating isolated mitochondria with cells (1 × 10^5^ cells/well of a 6-well plate) in 2 ml of standard medium at 37°C under 5% CO_2_ for 24 hrs. For analysing dose–response relationships, the total number of cells and DsRed-positive cells were calculated after 2 hrs co-incubation by using a Tali™ Image-Based Cytometer (Life Technologies) [18].

### Fluorescent staining and time-lapse microscopy

Immunofluorescence microscopy analysis was conducted by placing 40 μg of mitochondria isolated from H9c2 cells and 1 × 10^5^ EMCs in a 35-mm glass bottom dish (Iwaki, Tokyo, Japan) with 2 ml of standard medium. After 24 hrs co-incubation, the cells were fixed with 4% paraformaldehyde at 4°C for 5 min. and permeabilized with 0.1% Triton X-100 at room temperature for 20 min. in the presence of a protein-blocking solution consisting of PBS supplemented with 5% normal goat serum (DakoCytomation, Tokyo, Japan). The cells were incubated overnight with the following primary antibodies in PBS at 4°C: mouse anti-human mitochondria (diluted 1:1600; Abcam, Tokyo, Japan) and rabbit anti-red fluorescence protein (RFP; diluted 1:200; Abcam), or mouse anti-human mitochondria (diluted 1:1600; Abcam) and rabbit anti-TOM20 (diluted 1:100; Santa Cruz Biotechnology, Santa Cruz, CA, USA). They were washed extensively in PBS and incubated at room temperature for 30 min. with DyLight 594-conjugated goat antimouse secondary antibody (diluted 1:300; Santa Cruz Biotechnology) and Alexa Fluor 488-conjugated goat anti-rabbit secondary antibody (diluted 1:300; Life Technologies). The nuclei were counterstained with 4′,6-diamidino-2-phenylindole (DAPI; diluted 1:500; Wako, Tokyo, Japan) in PBS at room temperature for 45 min. To prevent fading during microscopy, the cells were mounted in DakoCytomation Fluorescent Mounting Medium (DakoCytomation). Immunofluorescence images were visualized and recorded by using a BIOREVO BZ-9000 fluorescence microscope (Keyence, Osaka, Japan). Cell 3D images were constructed from 30 pictures taken as 0.1-μm sections from the bottom to top of cells by using an automatic Z-axis motor. Deconvolution was applied for each image and the sequential images were stacked and colligated. For time-lapse video microscopy, 40 μg of isolated mitochondria from EMCs-DsRed2 mito were incubated with 1 × 10^5^ EMCs on 35-mm glass bottom dishes (Iwaki) in 2 ml of standard medium. RFP images and phase images were taken every 5 min. for 6 hrs from the beginning of co-incubation. During time-lapse recording, the environmental chamber was maintained at 37°C under 5% CO_2_ and humidified air (Tokai Hit, Shizuoka, Japan; *n* = 3).

### Transmission electron microscopy and immunoelectron microscopy

Isolated mitochondria (100 μg) were fixed with 2% paraformaldehyde (TAAB Laboratory Equipment Ltd., Aldermaston, UK) and 2% glutaraldehyde (Electron Microscopy Sciences, Hatfield, PA, USA) in 0.1 M cacodylate buffer (Electron Microscopy Sciences). The fixed samples were dehydrated through a series of graded ethanol (Wako). The samples were infiltrated with propylene oxide and embedded in a mixture of propylene oxide and resin (Nisshin EM, Tokyo, Japan). The samples were transferred to 100% resin and polymerized. Ultrathin sections (70 nm) were cut from the resin blocks by using a diamond knife mounted on an Ultracut (Leica, Tokyo, Japan). The sections were placed on copper grids, stained with 2% uranyl acetate (Merck, Darmstadt, Germany), rinsed with distilled water, followed by staining with Lead stain solution (Sigma-Aldrich).

EMCs co-incubated with isolated DsRed2-labelled mitochondria were examined by immunoelectron microscopy. A total of 20 μg of mitochondria were delivered to 2 × 10^5^ EMCs on a 24-well plate (Iwaki) in 500 μl of standard medium. The samples on the Mo grids were frozen and dehydrated through the anhydrous ethanol and infiltrated with a mixture of ethanol and resin. After embedding and polymerization, the blocks were ultra-thin sectioned at 80 nm. The sections on nickel grids were incubated with rabbit anti-RFP antibody (diluted 1:100; Abcam) for 90 min. at room temperature. They were washed extensively in PBS and incubated in gold-conjugated goat anti-rabbit secondary antibody (Abcam) for 1 hr at room temperature. The sections were stained with 2% uranyl acetate, rinsed with distilled water, followed by staining with Lead stain solution. The grids were visualized by transmission electron microscopy (JEOL, Tokyo, Japan) at an acceleration voltage of 80 kV. Digital images were acquired by using a CCD camera (Olympus, Tokyo, Japan).

### PCR for mtDNA

Specific primers for genomic PCR were designed to compare mtDNA and the nuclear DNA. The forward and reverse primer sequences were as follows, respectively: 5′-CCCTAAAACCCGCCACATCT-3′ and 5′-GAGCGATGGTGAGAGCTAAGGT-3′ for human NADH dehydrogenase subunit 1 (ND1); 5′-CACCCCCTTATCAACCT CAA-3′ and 5′-ATTTGTTTCTGCGAGGGTTG-3′ for rat ND1; 5′-TGCCCTAGACTTCGAGCAAGG-3′ and 5′-CGCTCATTGCCGATAGTGATG-3′ for rat actin; and 5′-CGAGTCGTCTTTCTCCTGATGAT-3′ and 5′-TTCTGGATTCCAATGCTTCGA-3′ for human lipoprotein lipase. For PCR analysis, DNA was extracted from EMCs, H9c2 cells and EMCs after 24 hrs co-incubation with mitochondria isolated from H9c2 cells by using a commercially available kit (Qiagen, Tokyo, Japan). The extracted DNA was subjected to selective amplification by PCR by using KOD FX Neo (Toyobo, Tokyo, Japan) under the following conditions: 35 cycles (98°C for 10 sec., 60°C for 30 sec. and 68°C for 30 sec.) after initial denaturation (94°C for 2 min.). Reaction specificity was verified by agarose gel electrophoresis on 2% gel (duplicate). Quantitative real-time PCR was performed with SYBR Premix Ex Taq (Takara, Tokyo, Japan) on a Thermal Cycler Dice Real Time System (Takara) under the following conditions: 40 cycles of PCR (95°C for 10 sec., 60°C for 1 min. and 72°C for 30 sec.) after initial denaturation (95°C for 2 min.). The amount of mtDNA was estimated from the content ratio of mtDNA:nuclear DNA and expressed relative to the content ratio of human mtDNA to human nuclear DNA in EMCs (*n* = 3, duplicate).

### Flow cytometric analysis

The cells were dispersed with 0.25% trypsin-EDTA and subjected to FACS analysis. The DsRed-positive cell population was evaluated by using 488 and 561 nm laser lines. Fluorescence data were collected by using SH800 (Sony, Tokyo, Japan). The flow cytometry files were analysed by using FlowJo software (TreeStar, San Carlos, CA, USA).

### Cell viability assay

Functional evidence for mitochondrial transfer was obtained by measuring cellular viability by using a resazurin-based assay kit (Promega, Tokyo, Japan), as described previously [19]. In brief, ρ0 cells were seeded in a 96-well plate (5 × 10^3^ cells/well) and co-incubated with 0, 2.5, 5, 10 and 25 μg/ml mitochondria isolated from EMCs at 37°C in 5% CO_2_. After a 24-hour co-incubation, the cells were washed and returned to the culture incubator until the assay was performed. On day 1, 3 and 7 after the co-incubation, resazurin dye was added to the wells and fluorescence was measured by using a plate reader with a 535-nm excitation filter and a 590-nm emission filter (Tecan, Tokyo, Japan; *n* = 5, duplicate).

### Measurements of cellular bioenergetics

An XF24 extracellular flux analyzer (Seahorse Biosciences, North Billerica, MA, USA) was used to measure cellular bioenergetic changes in ρ0 cells after mitochondrial transfer, as described previously [20]. In brief, ρ0 cells were seeded on XF24-well microplates (3 × 10^4^ cells/well) and co-incubated with 0, 6, 12 and 24 μg/ml mitochondria isolated from EMCs. EMCs were seeded on XF24-well microplates as positive control (3 × 10^4^ cells/well). After a 24-hour co-incubation, the cells were washed and returned to the culture incubator until the assay was performed. On day 3, the cells were washed twice and resuspended in 675 μl of unbuffered DMEM supplemented with 25 mM glucose, 4 mM glutamine and 1 mM sodium pyruvate (Life Technologies). The cells were equilibrated in a non-CO_2_ incubator for 60 min. prior to the assay. After three baseline measurements, oligomycin (1 μM), carbonyl cyanide p-trifluoromethoxyphenylhydrazone (FCCP, 2 μM) and rotenone/antimycin A (1 μM) were sequentially added to each well. Data were expressed as the oxygen consumption rates (OCR; pmol/min) or extracellular acidification rates (ECAR; mpH/min). Basal respiration, ATP production, maximal respiration and spare respiratory capacity were calculated, as described previously [21] (*n* = 5).

### Inhibition of mitochondrial transfer

The macropinocytosis inhibitor ethyl isopropyl amiloride (EIPA; Sigma-Aldrich) was used as described previously [22]. In brief, the EMCs (1 × 10^5^ cells/well of a 6-well plate) were pre-treated with 2 ml of standard medium containing 25 or 50 μM of EIPA at 37°C under 5% CO_2_ for 30 min. Then, 40 μg of mitochondria isolated from EMCs-DsRed2 mito were added and co-incubated at 37°C under 5% CO_2_ for 2 hrs. After 2 hrs co-incubation, the cells were subjected to live fluorescence imaging and FACS analysis (triplicate).

### Statistical analysis

Results are expressed as the mean ± SEM Statistical significance of multiple group differences was evaluated by anova followed by Dunnett's multiple comparison post hoc tests by using the Microsoft Office Excel application and the Statcel 3 program (OMS, Tokyo, Japan). *P* < 0.05 was considered significant.

## Results

### Isolation of mitochondria from cultured cells

**Fig. 1:**
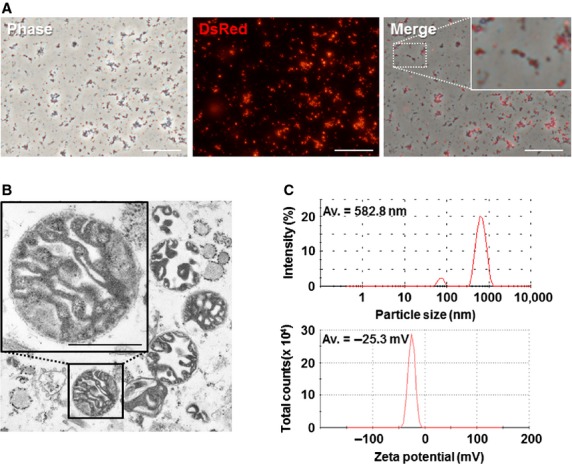
(**A**) Mitochondria isolated from EMCs-DsRed2 mito cells. Phase contrast image (left), fluorescence image (middle) and merged image (right). Scale bars, 50 μm. (**B**) Transmission electron microscopy of isolated mitochondria. Inset: a high magnification image of isolated mitochondria; scale bar, 500 nm. (**C**) Characterization of isolated mitochondria by Zetasizer. Particle size (upper graph) and zeta potential distribution (lower graph) were measured. Av, average.

Mitochondria were isolated from human uterine endometrial gland-derived mesenchymal cells (EMCs) carrying DsRed2-labelled mitochondria (EMCs-DsRed2 mito) to monitor mitochondrial transfer (Fig. S1). Fluorescence microscopic analysis showed that a DsRed-positive mitochondria-enriched fraction was obtained from EMCs-DsRed2 mito (Fig. 1A). Transmission electron microscopy revealed that this fraction contained morphologically intact mitochondria (Fig. 1B). To evaluate the profiles of this mitochondria-enriched fraction, particle size distribution and zeta potential were measured by using a Zetasizer. The particle sizes ranged from 141.8 to 5560.0 nm, with an average size of 582.8 nm (polydispersity index = 0.34). These particles had a negatively charged surface (−25.3 ± 6.4 mV; Fig. 1C).

### Identification of transferred xenogeneic mitochondria

**Fig. 2:**
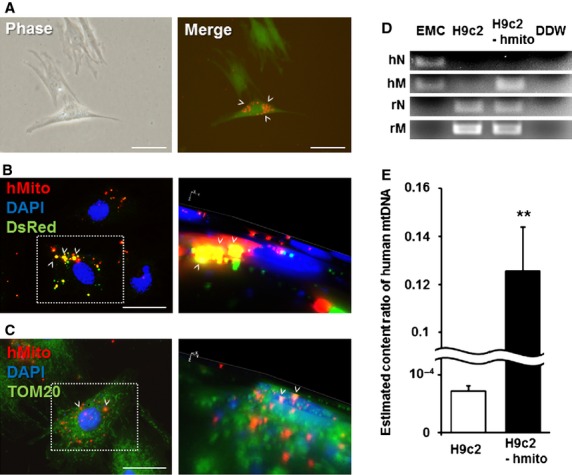
(**A**) Live images of GFP-expressing H9c2 cells containing DsRed2-labelled human mitochondria. Phase contrast image (left) and merged fluorescent image (DsRed and GFP; right). White arrow heads: transferred DsRed2-mitochondria; scale bars, 100 μm. (**B** and **C**) Immunofluorescent staining of the H9c2 cells transferred with human mitochondria. Merged fluorescence images with human mitochondria (red) and DsRed2 protein (green; **B**, left), or human mitochondria (red) and the mitochondrial outer membrane receptor TOM20 (green; **C**, left). Nuclei were stained with DAPI (blue). The right panels in B and C show 3D reconstructed images of the left panels. Left panel inset: focused sites of 3D reconstructed images. White arrow heads indicate transferred mitochondria; scale bars, 100 μm. (**D** and **E**) PCR analysis of the H9c2 cells transferred with human mitochondria. (**D**) Human mtDNA detection from the PCR products, and (**E**) the relative content ratio of human mtDNA to human nuclear DNA in EMCs. Error bars represent SEM. **Significantly different, *P* < 0.01. EMCs, Human uterine endometrial gland-derived mesenchymal cells; H9c2, rat H9c2 cardiomyoblasts; H9c2-hmito, H9c2 cells transferred with human mitochondria; DDW, distilled deionized water; hN, human nuclear DNA; hmito, human mtDNA; rN, rat nuclear DNA; rM, rat mtDNA.

DsRed2-labelled human mitochondria were isolated from EMCs-DsRed2 mito and immediately co-incubated for 24 hrs with rat H9c2 cardiomyoblasts or H9c2 stably expressing GFP to exclude the false positive result. Live fluorescence imaging suggested that the DsRed2-labelled mitochondria were internalized into the H9c2 cells (Fig. 2A). Immunohistochemical staining of these H9c2 cells with a human-specific mitochondrial antibody demonstrated that internalized human mitochondria colocalized with DsRed2 (Fig. 2B, left) and the mitochondrial outer membrane receptor TOM20 (Fig. 2C, left). The 3D reconstruction images of these immunohistochemically stained specimens confirmed that the human mitochondria were not located on the cell surface but within the cells (Fig. 2B, right and C, right and Fig. S4).

The PCR analysis of H9c2 cells transferred with human mitochondria demonstrated the presence of human mtDNA (Fig. 2D). In addition, quantitative real-time PCR confirmed the existence of human mtDNA in the H9c2 cells transferred with human mitochondria, which was estimated from the content ratio of mtDNA to nuclear DNA (Fig. 2E).

### Mitochondrial dynamics following transfer

**Fig. 3:**
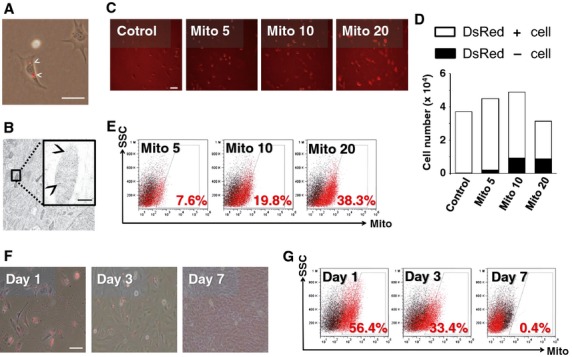
(**A**) Representative live fluorescence image of EMCs transferred with DsRed2-labelled human mitochondria. White arrow heads indicate the transferred mitochondria; scale bar, 100 μm. (**B**) Immunoelectron microscopy of EMCs transferred with DsRed2-labelled human mitochondria. Inset: high magnification image of the transferred DsRed2-labelled mitochondria. Black arrow heads indicate DsRed protein; scale bar, 100 nm. (**C**–**E**) Dose-dependent increase in the percentage of DsRed-positive cells. (**C**) Representative live fluorescence images of transferred cells; scale bar, 200 μm. (**D**) Total number of cells and mitochondria-transferred cells using image-based cytometer analysis. Black and white bar sections indicate cells with and without exogenous mitochondria, respectively. (**E**) FACS analysis of mitochondria-transferred cells. Controls (cells without mitochondrial transfer) are represented by the black dots, and cells with mitochondrial transfer by the red dots Control, no mitochondria delivery; Mito 5–20, 5–20 μg/ml of mitochondria delivery. (**F** and **G**) Time course of transferred mitochondria. (**F**) Representative live fluorescence images and FACS analysis of transferred cells on day 1, 3 and 7 after mitochondrial transfer; scale bar, 100 μm. (**G**) Control (cells without mitochondrial transfer) are represented by the black dots, and cells with mitochondrial transfer by the red dots.

After co-incubation of the recipient EMCs with isolated DsRed2-labelled mitochondria derived from homogeneic EMCs for 24 hrs, live fluorescence imaging showed DsRed2-labelled mitochondria internalized into the EMCs (Fig. 3A). Immunoelectron microscopy performed with anti-DsRed antibody and gold-conjugated secondary antibody confirmed that DsRed2-labelled mitochondria were present within the EMCs (Fig. 3B).

The optimal dose of mitochondria for the transfer procedure was determined from dose–response relationships analysed by image-based cytometer analysis. The EMCs were co-incubated with 0, 5, 10 or 20 μg/ml of isolated DsRed2-labelled mitochondria. The percentage of DsRed-positive cells increased in a dose-dependent manner, with 0.5%, 4.3%, 18.9% and 27.6%, respectively (Fig. 3C and D). Fluorescence-activated cell sorting analysis generated comparable results, with 0.3%, 7.6%, 19.8% and 38.3%, respectively (Fig. 3E). Furthermore, the number of total EMCs co-incubated with 20 μg/ml of isolated mitochondria decreased compared with that co-incubated with fewer mitochondria (Fig. 3D). The persistence of the exogenous mitochondria in the recipient cells was investigated for 7 days after 24 hrs co-incubation of EMCs with 20 μg/ml of isolated mitochondria. Live fluorescence imaging (Fig. 3F) and FACS analysis (Fig. 3G) showed that the number of cells containing DsRed2-positive mitochondria declined over time and disappeared within a week.

### Recognition and engulfment of exogenic mitochondria

**Fig. 4:**
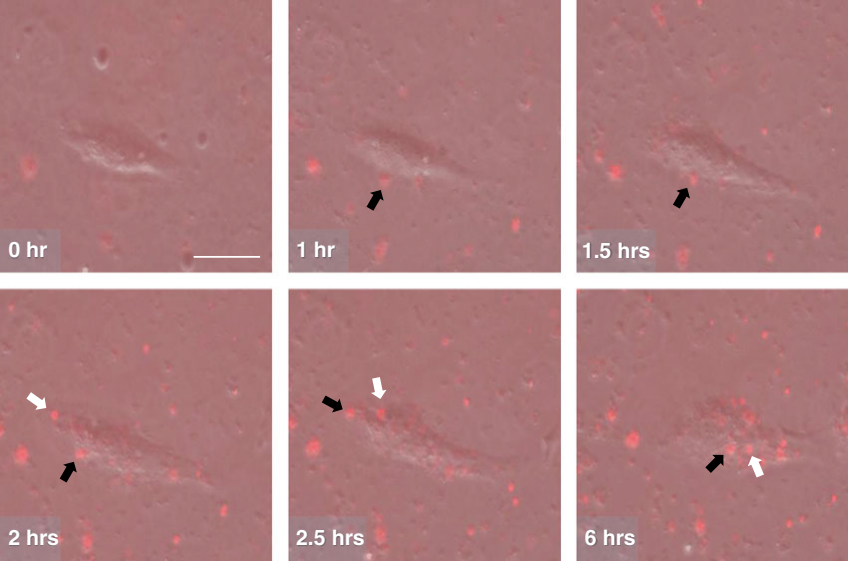
Time-lapse fluorescent microscopy images of mitochondrial transfer. The numbers are time elapsed (h) since the initiation of co-incubation. The black and white arrows indicate the same mitochondria; scale bar, 100 μm. The movie file is supplemented in Video S1.

To capture the dynamic behaviour of mitochondrial transfer, time-lapse microscopy was recorded during co-incubation of EMCs with 20 μg/ml of isolated DsRed2-labelled mitochondria. The images revealed that mitochondrial transfer occurred within 1–2 hrs after the initiation of co-incubation. The cells seemed to recognize and engulf actively exogenic mitochondria attached to their surfaces. Subsequently, the internalized mitochondria accumulated in the perinuclear region (Fig. 4 and Video S1).

### Functional gain from exogenous mitochondria

**Fig. 5:**
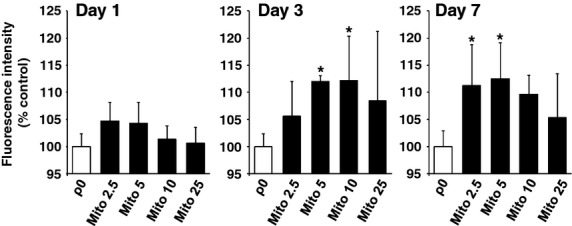
Cell viability assay in mitochondria-transferred ρ0 cells on day 1, 3 and 7 after mitochondrial transfer. ρ0, no mitochondria delivery; Mito 2.5–25, 2.5–25 μg/ml of mitochondria delivery. Error bars represent standard error of the mean. *Significantly different, *P* < 0.05.

To assess the functional impact of mitochondrial internalization on the recipient cells, resazurin-based assays were performed in ρ0 cells after co-incubation of the with 0, 2.5, 5, 10 and 25 μg/ml of isolated mitochondria (MITO-ρ0). One day after mitochondrial transfer, no significant difference was observed in cellular viability between the groups (Fig. 5, left). On day 3, the MITO-ρ0 at the dose of 5 or 10 μg/ml mitochondria exhibited a significant recovery in cellular viability compared with the control ρ0 cells (Fig. 5, middle). On day 7, incubation with 2.5 or 5 μg/ml mitochondria also generated significant recovery (Fig. 5, right). In contrast, co-incubation with 25 μg/ml mitochondria worsened the viability in the ρ0 cells. This overdose effect was consistent with the image-based cytometer analysis (Fig. 3D). UV-treated mitochondria failed to demonstrate significant increase in cellular viability (Fig. S5).

**Fig. 6:**
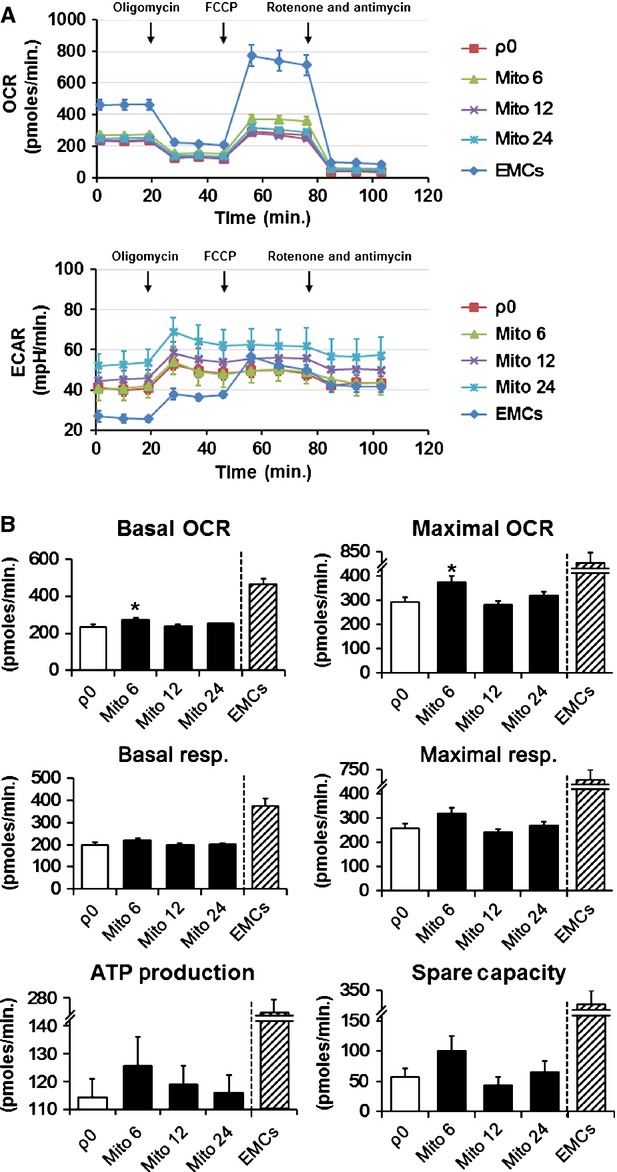
Measurements of cellular bioenergetics after mitochondrial transfer. (**A**) Investigation of mitochondrial function in terms of oxygen consumption rate (OCR) and extracellular acidification rate (ECAR) in mitochondria-transferred ρ0 cells on day 3 after mitochondrial transfer. Black arrows indicate the time of addition of each mitochondrial functional modifier. ρ0, no mitochondrial delivery; Mito 6–24, 6–24 μg/ml mitochondria delivery; EMCs, human uterine endometrial gland-derived mesenchymal cells. Data are expressed as mean ± standard error. (**B**) Basal OCR, maximal OCR, basal respiration, maximal respiration, adenosine triphosphate (ATP) production and spare respiratory capacity (spare capacity) of the cells. Error bars represent standard error. *Significantly different, *P* < 0.05.

To evaluate the effect of mitochondrial transfer on mitochondrial function, cellular bioenergetics were measured in ρ0 cells after co-incubation with 0, 6, 12 and 24 μg/ml mitochondria isolated from EMCs (MITO-ρ0). Three days after the transfer, the basal and maximal OCRs of mito-ρ0 cells were significantly improved at the mitochondrial concentration of 6 μg/ml, without altering the ECARs (Fig. 6A). In contrast, higher concentrations of mitochondria failed to produce a significant enhancement of mitochondrial function in ρ0 cells. Further analyses revealed that spare respiratory capacity markedly increased by 73.8 ± 13.4% in ρ0 cells co-incubated with 6 μg/ml mitochondria. In contrast, basal respiration, ATP production and maximal respiration slightly increased by 10.1 ± 5.4%, 10.0 ± 5.7% and 24.3 ± 7.5%, respectively. However, these variables did not show a significant difference.

### Mechanism of mitochondrial uptake

**Fig. 7:**
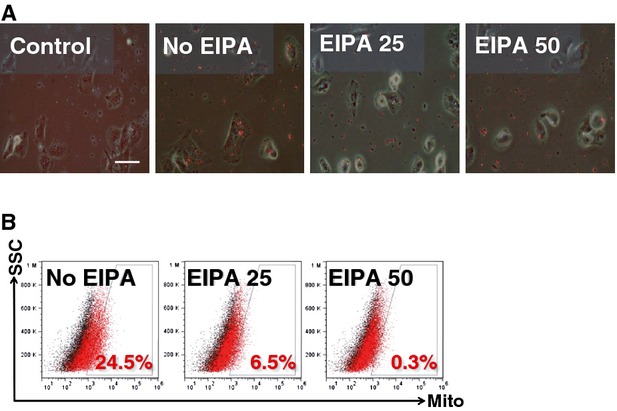
Impact of the macropinocytosis inhibitor EIPA on mitochondrial transfer. (**A**) Representative live fluorescence images and (**B**) FACS analysis for mitochondrial transfer performed in the presence of EIPA. Scale bar, 100 μm. (**B**) Cells without mitochondrial transfer are represented by the black dots and cells with mitochondrial transfer by the red dots. Control, no mitochondria delivery No EIPA, no EIPA treatment; EIPA 25, 25 μM EIPA treatment; EIPA 50, 50 μM EIPA treatment.

To elucidate the mechanism of mitochondrial transfer, we used EIPA as one of the specific inhibitors of macropinocytosis as described previously [22]. The co-incubation of EMCs with isolated DsRed2-labelled mitochondria (2 hrs) was performed in the presence of EIPA (25 or 50 μM). Subsequently, fluorescence microscopy clearly showed a suppression of mitochondrial transfer in the EIPA group compared with the no-EIPA group (Fig. 7A). FACS analysis confirmed that EIPA caused a significant decrease in the percentage of DsRed-positive cells (6.5% with 25 μM and 0.3% with 50 μM) compared with the no-EIPA group (24.5%; Fig. 7B). Furthermore, EIPA treatment abolished the therapeutic effect of mitochondrial transfer in ρ0 cells (Fig. S5). To further assess the contribution of macropinocytosis in mitochondrial transfer, the inhibitory effects of other macropinocytosis and endocytosis inhibitors were measured by FACS after co-incubation of EMCs with isolated DsRed2-labelled mitochondria. Mitochondrial transfer was reduced by cytochalasin D (inhibitor of actin polymerizaton) and nocodazole (inhibitor of microtubule assembly) but not by chlorpromazine (inhibitor of clathrin-mediated endocytosis; Fig. S6).

## Discussion

In this study, we demonstrated that isolated mitochondria are internalized into cells by simple co-incubation by using genetically labelled mitochondria. Isolated mitochondria were internalized into homogeneic and xenogeneic cells within a few hours of co-incubation. Furthermore, we observed how mitochondria are engulfed by the recipient cells and how they behave inside the cells after internalization by time-lapse video microscopy. We also showed that optimal mitochondrial internalization significantly improves the mitochondrial function in mtDNA-depleted cells, and that these benefits lasted several days. Finally, we demonstrated the possible involvement of macropinocytosis in this process by using various inhibitors of endocytosis.

The cellular uptake of isolated mitochondria was originally reported in 1982 by Clark and Shay [9]. They reported that isolated mitochondria labelled with a mitochondria-specific dye accumulated in cells. Furthermore, the cells sensitive to chloramphenicol (CAP) were transformed into resistant cells after co-incubation with isolated mitochondria carrying mutant mtDNA conferring resistance to CAP. Finally, they reported that this phenomenon was not observed with xenogeneic mitochondria. Later, Ber *et al*. confirmed that a hamster cell line transformed to CAP resistant after a similar co-incubation [23]. In addition, they showed by electron microscopy that isolated mitochondria were engulfed by recipient cells at 37°C but not at 4°C. After a long inactive period in the scientific literature, Katrangi *et al*. reported that isolated mouse mitochondria labelled with a mitochondria-specific dye accumulated in human and mouse cell lines after co-incubation [10]. They provided evidence that mouse mtDNA was detected by human ρ0 cells and that cellular uptake of isolated mitochondria led to an increase in oxygen consumption. However, Chang *et al*. recently reported that cellular uptake was only observed when the isolated mitochondria were treated with the cell-penetrating peptide Pep-1 [11]. Similarly, Spees *et al*. claimed that isolated mitochondria did not accumulate in the cells after simple co-incubation [6]. In the present study, we clearly demonstrated that isolated mitochondria were transferred into the recipient cells during simple co-incubation by using genetically labelled mitochondria, which prevented the leakage of the mitochondria staining dye. We actually observed that the mitochondria of the recipient cells were stained by the dye leaking from the dye-labelled isolated mitochondria after co-incubation (*data not shown*).

We confirmed that human mitochondria are transferred into rat cells as well as human cells. However, a complete set of cognate chromosomes was necessary for the maintenance of mitochondrial genomes and functions [24]. Mitochondria have co-evolved with their host cells for ages, that is supposed to lead to species-specific compatibility [25] . Therefore, we only conducted interspecies mitochondrial transfer to validate the process and investigated the therapeutic potential of the process by using homogeneic mitochondria.

In previous studies, exogenous mitochondria were maintained in the recipient cells for longer periods [26,27]. In contrast, our study demonstrated that the internalized mitochondria disappeared within a week. In addition, transmission electron microscopy showed that some exogenous DsRed-labelled mitochondria were identified in the autophagosomes after mitochondrial transfer within recipient cells (Fig. S6). Exogenous mitochondria may be selectively degraded after mitochondrial transfer.

In our study, mitochondrial transfer significantly improved the mitochondrial functions of the ρ0 cells. Likewise, the transfer of oocyte content from fertile donor's egg containing mitochondria to the ooplasm of recipient's egg improved the outcome of implantation after *in vitro* fertilization [28,29]. Moreover, the introduction of normal mitochondria into eggs carrying mutated mtDNA appeared to be a promising tool for the treatment of human hereditary mitochondrial disorders [26,30]. It is a widely accepted theory that mitochondrial dysfunction is associated with many human diseases, such as cancer, ageing, and metabolic, cardiovascular and neurodegenerative diseases [7,8,31,32]. We showed that a single dose of isolated mitochondria led to a significant improvement in viability and mitochondrial respiratory function in the recipient cells and the beneficial effects lasted for several days. These data are consistent with an *in vivo* study showing that bone marrow-derived stromal cells exerted a protective effect against bacterial endotoxin-induced lung injury through the transfer of mitochondria from donor cells [4]. Even though different cell types and mitochondrial sources were used for the experiments, our results shared striking similarities to previous studies by Masuzawa and McCully [13,19]. They also demonstrated that isolated mitochondria were internalized into recipient cells within 2–24 hrs. These mitochondria were localized in the perinuclear region, were maintained in the recipient cells and were involved in ATP synthesis and enhancement of cellular viability. These findings suggested that direct mitochondrial transfer and their beneficial effects are probably a universal biological phenomena in mammalian cells. Taken together, these data illustrate the potential of mitochondrial transfer for the treatment of diseases associated with mitochondrial dysfunction, such as myocardial infarction and stroke.

Isolated mitochondria are anticipated to be difficult to internalize into cells because of their relatively large size and negatively charged surface. However, interestingly, a simple co-incubation of isolated mitochondria with recipient cells resulted in mitochondrial internalization. Therefore, cells must possess the inherent ability to detect and uptake extracellular mitochondria. While considerable evidence supports the endosymbiotic theory that mitochondria in eukaryotic cells originate from symbiosis between separate organisms, there is an ongoing debate on the mechanism [25]. Elucidating the precise mechanism of direct mitochondrial transfer may uncover the evolutionary history of eukaryotes. In this regard, the suppression of mitochondrial transfer by EIPA (inhibitor of the Na^+^/H^+^ exchange), nocodazole (inhibitor of microtubule assembly) and cytochalasin D (inhibitor of actin polymerization), but not by chlorpromazine (inhibitor of clathrin-mediated endocytosis), suggested a possible involvement of macropinocyotosis in direct mitochondrial transfer [33]. However, the sub-classification of macropinocytosis is still under debate and an endocytotic pathway implies that the mitochondria would have to escape the endosomes to perform their function.

In this study, the isolated mitochondria-enriched fraction was used for experiments. This fraction also contained various intracellular organelles such as damaged and dysfunctional mitochondria that are proposed to be of no benefit and even harmful to the host cells [34]. A previous study has also indicated that freshly isolated, intact, viable and respiration-competent mitochondria were required to exert a therapeutic effect [13]. Our experiments also revealed that UV-treated mitochondria failed to improve cellular viability in recipient cells. To investigate the authentic effect of direct mitochondrial transfer, we need more sophisticated and minimally invasive methods to sort out the functionally intact mitochondria from the crude cell extracts. Moreover, our study also revealed the possible cytotoxicity of mitochondrial internalization in case of overdose. The development of less invasive delivery methods may be required to exert profound beneficial effects of mitochondrial transfer.

In conclusion, this multidisciplinary study provides quantitative and imaging evidence that the direct transfer of exogenous mitochondria into mammalian cells constitutes a promising approach for the treatment of various diseases associated with mitochondrial dysfunction.

## Supporting information

**Figure S1** pMXs-DsRed2-mito. **Figure S2** Correlation between the number of isolated mitochondria and protein concentration. **Figure S3** Exogenous mitochondria in autophagosome. **Figure S4** Immunofluorescent staining of the H9c2 cells transferred with human mitochondria. **Figure S5** Cell viability assay in mitochondria-transferred ρ0 cells. **Figure S6** Effect of inhibitors of macropinocytosis and endocytosis on mitochondrial transfer. **Figure S7** Immunofluorescent staining of fixed cells with Anti-DsRed antibody. **Figure S8** Flowcytometric analysis of membrane potential in isolated mitochondria.

**Video S1** Time-lapse fluorescent microscopy images of mitochondrial transfer.
