# Supplementary material for: Internalization of isolated functional mitochondria: involvement of macropinocytosis
Source: J Cell Mol Med. 2014 Jun 9;18(8):1694–703. doi: 10.1111/jcmm.12316 (PMC4190914; doi:10.1111/jcmm.12316)
Supplement: Supplementary file 1 [file jcmm0018-1694-SD1.docx]

**
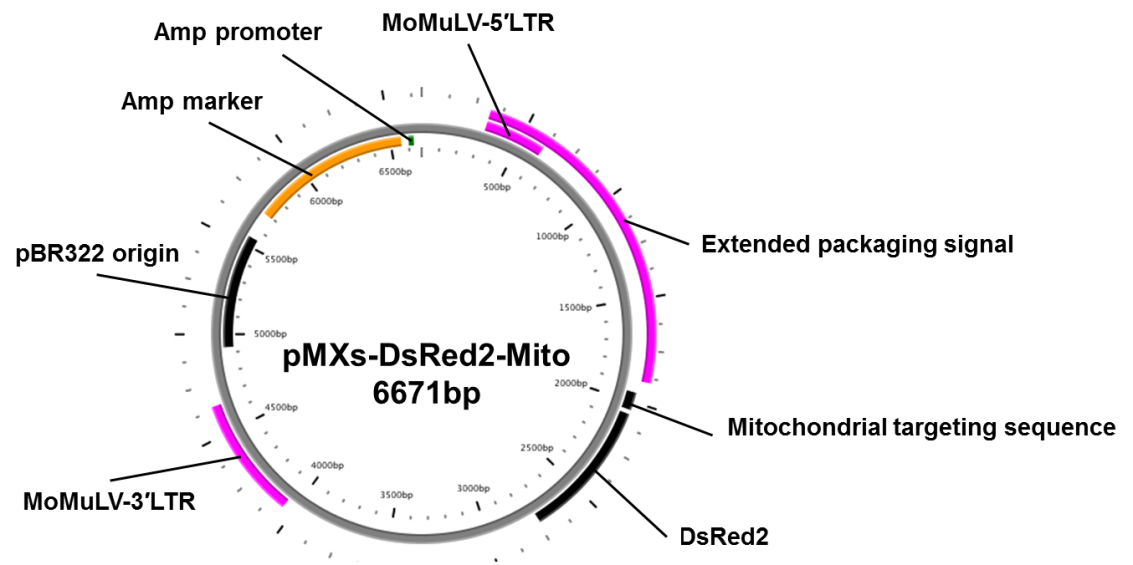
Supplementary Figure 1; pMXs-DsRed2-mito.**

**
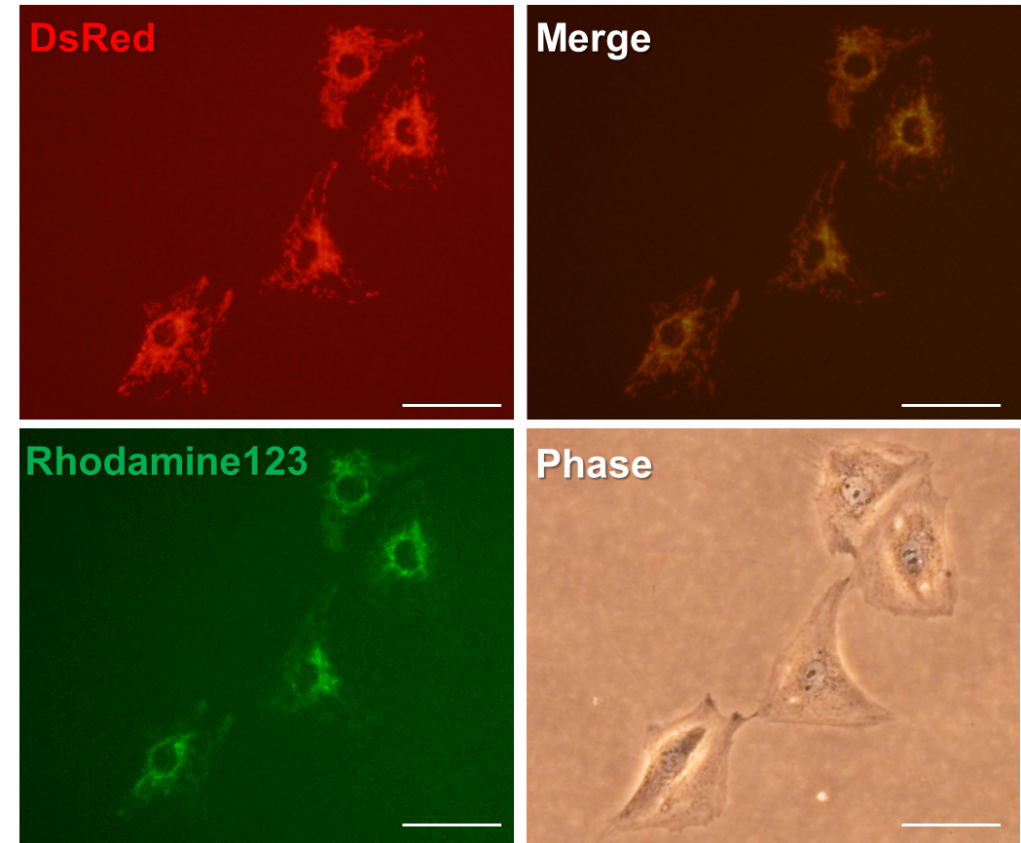
**

**(Top)** Plasmid map of pMXs-DsRed-mito. The correct targeting of Mito-DsRed2 fusion protein to the host cell’s mitochondria was previously confirmed [[1](#_ENREF_1)]. MoMuLV-5′LTR (319,614), Extended packaging signal (319,1896), Mitochondrial targeting sequence (1954,2041), DsRed2 (2062,2740), MoMuLV-3′LTR (4053,4645), pBR322 origin (4931,5550), Amp marker (5698,6558), Amp promoter (6600,6628).

**(Bottom)** Human uterine endometrial gland-derived mesenchymal cells (EMCs), carrying DsRed2-labeled mitochondria stained with rhodamine123 (2 μm, Life Technologies™). After 10-min incubation, cells were washed twice and subjected to live fluorescence imaging. The merged image confirmed that the DsRed protein was correctly delivered to mitochondrial foci. DsRed (upper left), Rhodamine123 (lower left), phase contrast image (lower right), and merged image (upper right). Scale bar, 100 μm.


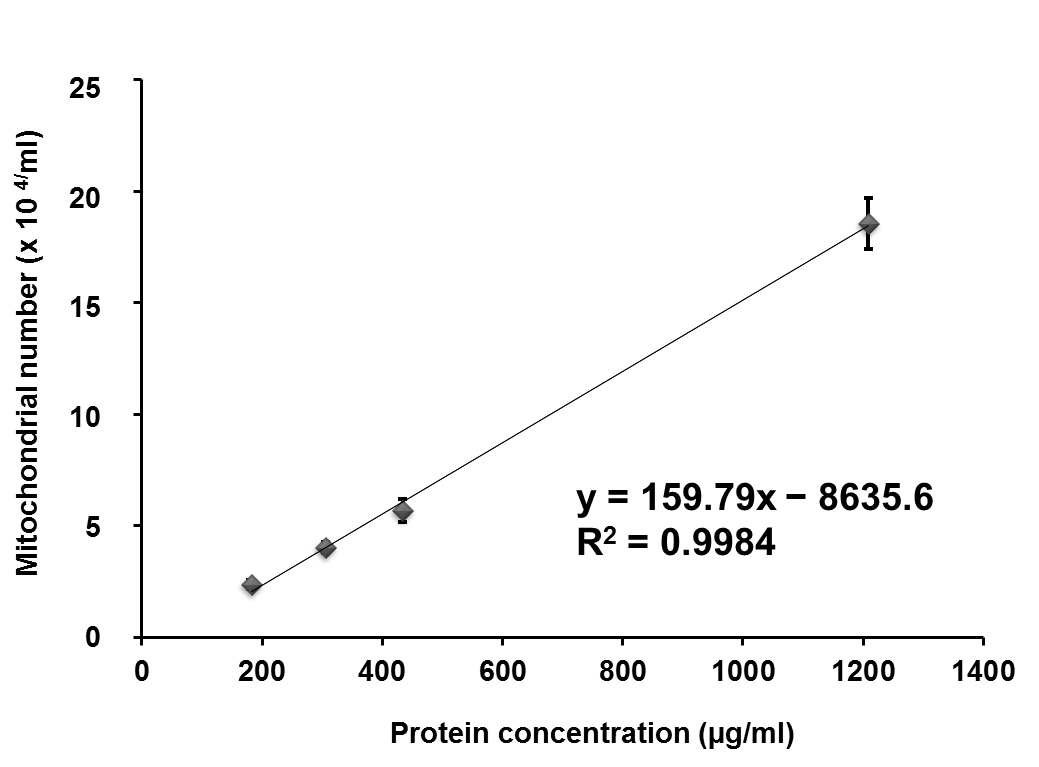
**Supplementary Figure 2; Correlation between the number of isolated mitochondria and protein concentration.**

The isolated mitochondria were resuspended in the homogenization buffer. Serial dilutions of this preparation were examined by a Bio-Rad protein assay kit and fluorescence imaging. The number of DsRed positive dots was counted as the number of isolated mitochondria per field in five randomly selected microscope fields. There was a good correlation between protein concentration of the mitochondria-enriched fraction and the number of isolated mitochondria (n = 3).

**Supplementary Figure 3; Exogenous mitochondria in autophagosome.**


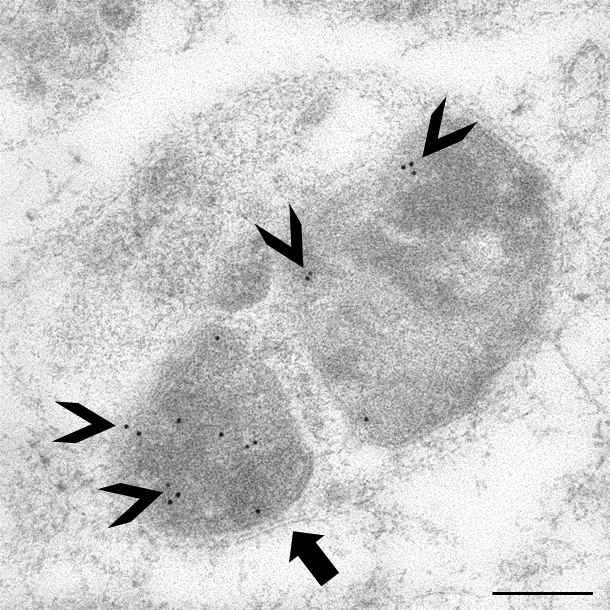


Immunoelectron microscopy of EMCs transferred with DsRed2-labeled human mitochondria. Some exogenous mitochondria were identified in the autophagosomes after mitochondrial transfer within the recipient cells. The black arrow indicates a double-membrane structure of the autophagosomes containing undigested cytoplasmic materials. The black arrow heads indicate DsRed protein. Scale bar, 200 nm.

**Supplementary Figure 4; Immunofluorescent staining of the H9c2 cells transferred with human mitochondria.**

**
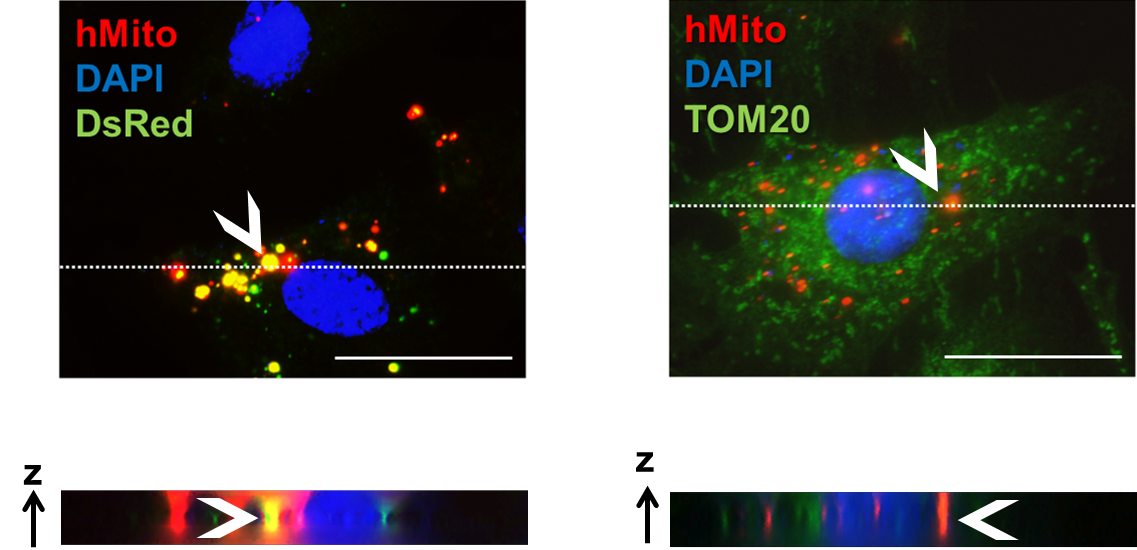
**

The images in 2B and 2C were projected along the z axis, respectively. The horizontal white break lines in the top images show the cutting section where the bottom images were obtained. The transferred mitochondria (white arrow heads) and the recipient nucleus are located at the same depth. The nuclei were stained with DAPI (blue). Scale bar, 100 mm.

**Supplementary Figure 5; Cell viability assay in mitochondria-transferred ρ0 cells**
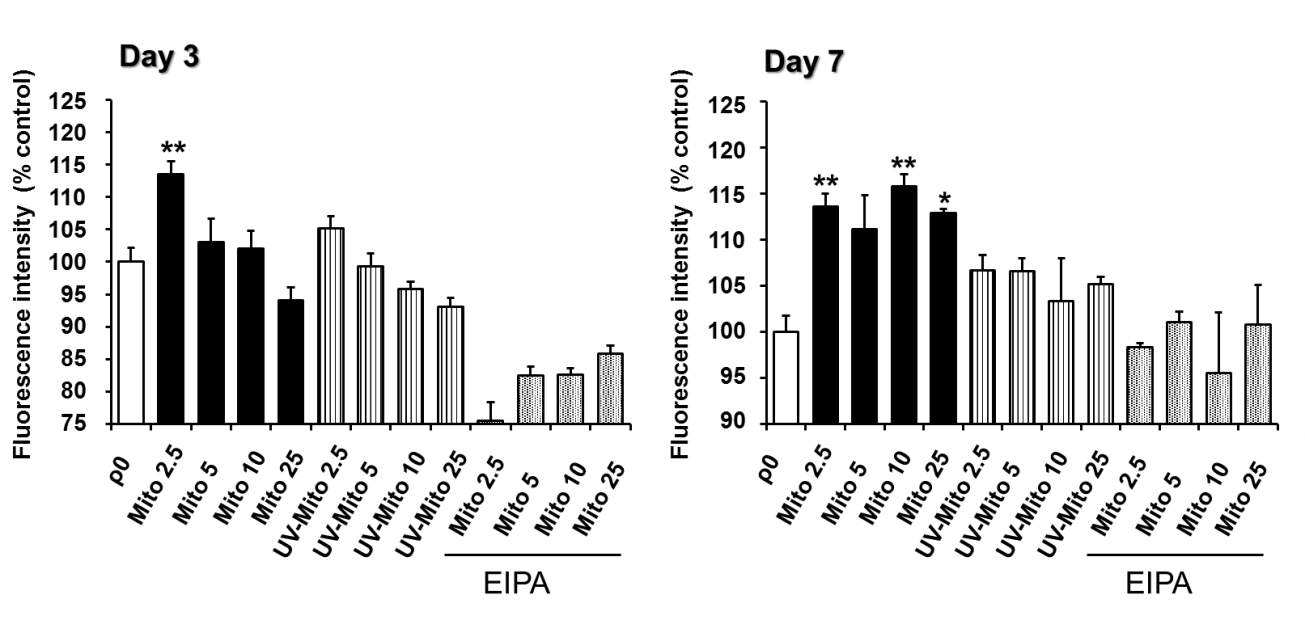


The cell viability assays were performed in mitochondria-transferred ρ0 cells on days 3 and 7 after mitochondrial transfer. As comparison groups, the cells after the co-incubation with the isolated mitochondria treated by overnight UV irradiation and the cells pretreated with EIPA (25 μm) and co-incubated with isolated mitochondria were examined (n = 5, duplicate). The UV treated mitochondria failed to improve ρ0 cell viability. The EIPA treatment abolished the effect of mitochondrial transfer. ρ0, no mitochondria delivery; Mito 2.5–25, 2.5–25 μg/ml of mitochondria delivery; UV-Mito 2.5-25, 2.5–25 μg/ml of UV treated mitochondria delivery. Error bars represent standard error of the mean. *Significantly different, *P* < 0.05. **Significantly different, *P* < 0.01.

**Supplementary Figure 6; Effect of inhibitors of macropinocytosis and endocytosis on mitochondrial transfer.**

**
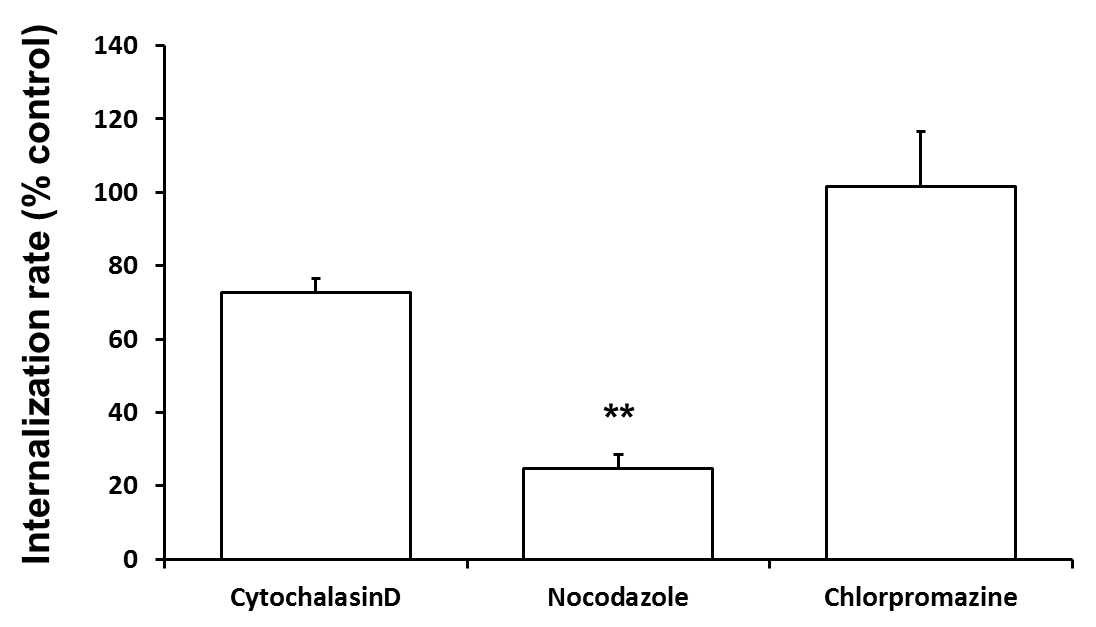
**

EMCs (1 × 10^5^ cells/well of a 6-well plate) were pretreated with 2 ml of standard medium containing 2 µm of cytochalasin D (Wako), 10 µM of nocodazole (Sigma), or 10 µm of chlorpromazine (Wako) at 37°C under 5% CO2 for 30 min [[2](#_ENREF_2)]. Then, 40 μg of mitochondria isolated from EMCs-DsRed2 mito were added and coincubated at 37°C under 5% CO2 for 2 h. After 2 h coincubation, the cells were subjected to flowcytometric analysis. The internalization rates were compared with mitochondria-transferred cells without any inhibitors. The mitochondrial transfer was reduced by cytochalasin D (inhibitor of actin polymerization), nocodazole (inhibitor of microtuble assembly), but not by chlorpromazine (inhibitor of clathrin mediated endocytosis). **Significantly different, *P* < 0.01.

**Supplementary Figure 7; Immunofluorescent staining of fixed cells with Anti-DsRed antibody.**

**
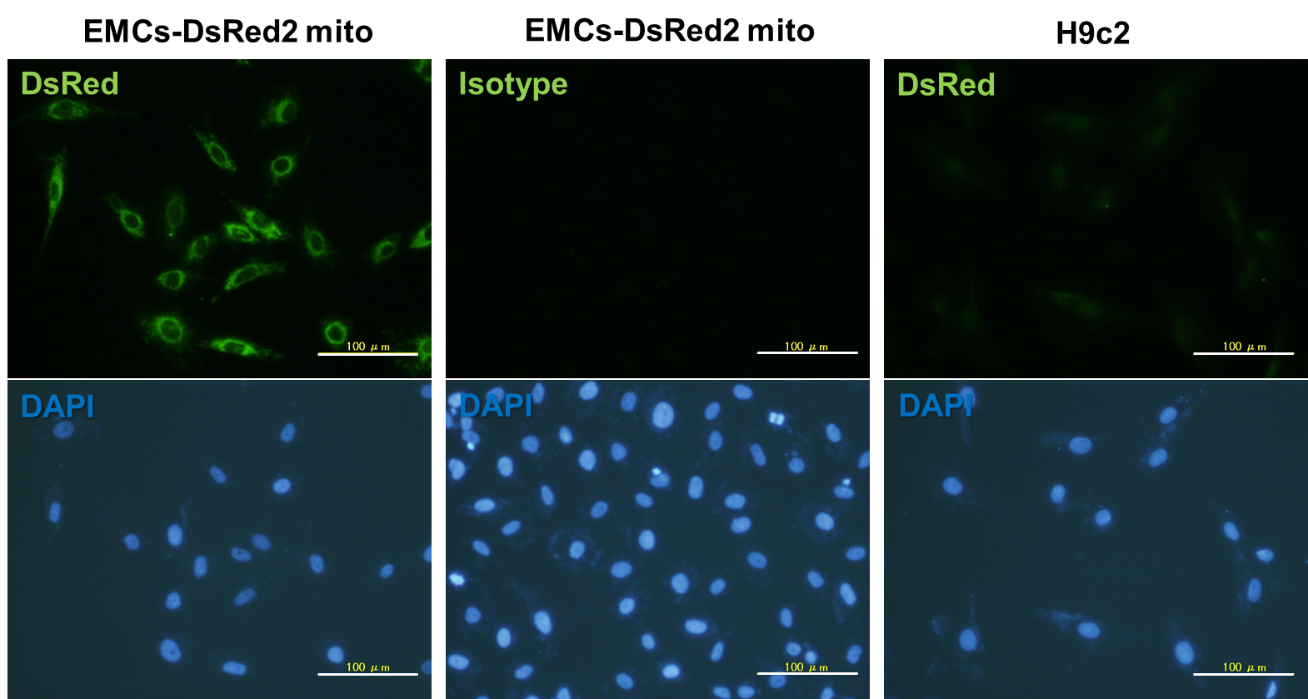
**

Anti-DsRed antibody specificity was confirmed in 4% paraformaldehyde-fixed EMCs carrying DsRed labeled mitochondria and H9c2. Rabbit anti-red fluorescence protein (RFP) (diluted 1:200; Abcam) was used at a 1:200 dilution, according to the manufacturer’s protocol. Rabbit polyclonal IgG were used as isotype control. An alexa Fluor 488-conjugated goat anti-rabbit secondary antibody was used as secondary antibody at a 1:300 dilution (green). Nuclei were stained with DAPI (blue). Scale bar, 100 mm. EMCs-DsRed2 mito, human uterine endometrial gland-derived mesenchymal cells carrying DsRed2-labeled mitochondria.


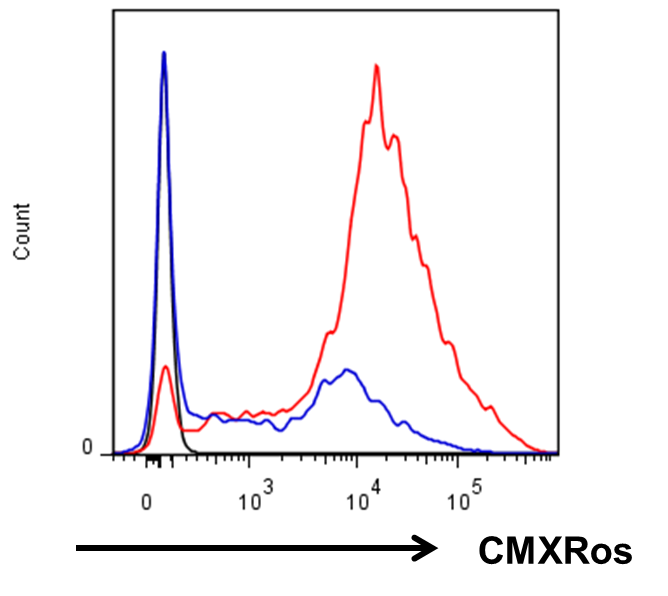
**Supplementary Figure 8; Flowcytometric analysis of membrane potential in isolated mitochondria.**

The mitochondria membrane potential was examined using MitoTracker Red CMXRos (Life Technologies™). Isolated mitochondria were stained with MitoTracker Red CMXRos (5 μm) for 10 min, as previously described [[3](#_ENREF_3)]. Flow cytometric analysis showed that the isolated mitochondria maintained the membrane potential. The pretreatment of isolated mitochondria with the uncoupler of oxidative phosphorylation FCCP caused large depolarization [[4](#_ENREF_4)]. Black curve, unstained mitochondria; red curve, CMXRos stained mitochondria; blue curve, CMXRos stained mitochondria after FCCP treatment.

1. **Romanello V, Guadagnin E Fau - Gomes L, Gomes L Fau - Roder I, Roder I Fau - Sandri C, Sandri C Fau - Petersen Y, Petersen Y Fau - Milan G, Milan G Fau - Masiero E, Masiero E Fau - Del Piccolo P, Del Piccolo P Fau - Foretz M, Foretz M Fau - Scorrano L, Scorrano L Fau - Rudolf R, Rudolf R Fau - Sandri M, Sandri M.** Mitochondrial fission and remodelling contributes to muscle atrophy. *EMBO J*. 2010; 29: 1774-85.

2. **Kee SH, Cho EJ, Song JW, Park KS, Baek LJ, Song KJ.** Effects of endocytosis inhibitory drugs on rubella virus entry into VeroE6 cells. *Microbiol Immunol*. 2004; 48: 823-9.

3. **McCully JD, Cowan DB, Pacak CA, Toumpoulis IK, Dayalan H, Levitsky S.** Injection of isolated mitochondria during early reperfusion for cardioprotection. *Am J Physiol Heart Circ Physiol*. 2009; 296: H94-H105.

4. **Cottet-Rousselle C, Ronot X, Leverve X, Mayol JF.** Cytometric assessment of mitochondria using fluorescent probes. *Cytometry A*. 2011; 79: 405-25.
